# Development of a predictive model and nomogram using CTPA parameters for the identification of group 1 pulmonary arterial hypertension

**DOI:** 10.1016/j.isci.2026.117763

**Published:** 2026-10-08

**Authors:** Huaxin Yuan, Iokfai Cheang, Gengmin Liang, Xu Zhu, Yutong Xing, Wenting Li, Shengen Liao, Jian He, Yunshan Cao, Xinli Li, Yi Xu, Yanli Zhou

**Affiliations:** 1 State Key Laboratory for Innovation and Transformation of Luobing Theory, Department of Cardiology, the First Affiliated Hospital with Nanjing Medical University, Jiangsu Province Hospital, Nanjing 210029, China; 2 Department of Cardiology, Luodian Hospital, Baoshan District, Shanghai 201908, China; 3 Department of Radiology, the First Affiliated Hospital with Nanjing Medical University, Jiangsu Province Hospital, Nanjing 210029, China; 4 Heart, Lung and Vessels Center, Sichuan Provincial People’s Hospital, University of Electronic Science and Technology of China, Chengdu 610072, China

**Keywords:** pulmonary hypertension, computed tomography pulmonary angiography, CTPA, predictive model, nomogram, right heart catheterization, RHC

## Abstract

This study aimed to identify computed tomography pulmonary angiography (CTPA)-derived predictors of pulmonary arterial hypertension (PAH) and to develop a prediction model and nomogram for non-invasive diagnosis. We retrospectively analyzed CTPA datasets from 203 patients in the training cohort and 98 patients in the validation cohort, stratified into PAH (confirmed by right heart catheterization) and non-PAH groups. Pulmonary vascular and cardiac structural metrics were measured. The least absolute shrinkage and selection operator (LASSO) and logistic regression identified interventricular septal angle (IVSA), interventricular septal thickness (IVST), and right ventricular outflow tract wall thickness (RVOTW) as independent predictors. The model achieved an area under the receiver operating characteristic curve (AUC) of 0.964 and 0.969 in the training and validation cohorts, respectively, with excellent calibration and clinical utility. The CTPA-based predictive model and nomogram may serve as a useful reference tool, potentially assisting in the identification of PAH and optimizing clinical workflows.

## Highlights

• A useful prediction model based on CTPA image was developed for group 1 PAH
• IVSA, IVST, and RVOTW were identified as the key predictors integrated into the model
• The model showed good performance with AUCs of 0.964 (training) and 0.969 (validation)
• The model aids in non-invasive PAH identification and might optimize clinical workflows

## Graphical abstract

**Figure:**
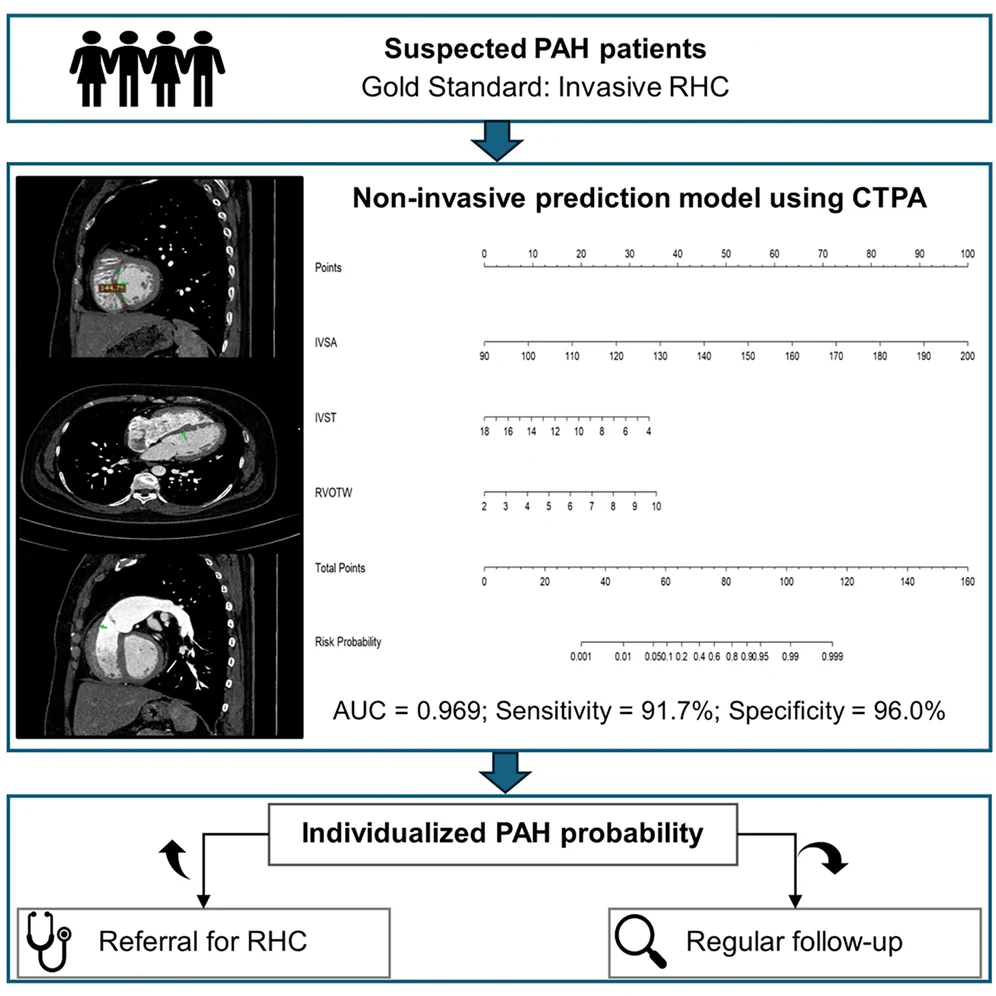

## Teaser

Health sciences; Artificial intelligence

## Introduction

Pulmonary arterial hypertension (PAH), a progressive and life-threatening disorder, is defined by right heart catheterization (RHC) as mean pulmonary artery pressure (mPAP) > 20 mmHg, pulmonary capillary wedge pressure (PCWP) ≤ 15 mmHg, and pulmonary vascular resistance (PVR) > 2 Wood units at rest. PAH is characterized by pulmonary vascular remodeling, increased pulmonary vascular resistance, and eventual right ventricular (RV) failure, leading to significant morbidity and mortality.^1^^,^^2^^,^^3^ Early identification and timely intervention are critical for improving prognosis; however, diagnosis remains challenging due to nonspecific symptoms and the reliance on RHC, the gold standard for hemodynamic confirmation.^4^^,^^5^ RHC is invasive, costly, and not universally accessible, creating barriers to timely diagnosis and management. These limitations highlight the urgent need for non-invasive tools to facilitate early detection, risk stratification, and triage of PAH.

Computed tomography pulmonary angiography (CTPA), a widely available and non-invasive imaging modality, has emerged as a valuable tool for evaluating PAH. CTPA provides detailed anatomical insights into pulmonary arterial morphology and RV structure, both of which are central to PAH pathophysiology.^6^^,^^7^^,^^8^ Key CTPA-derived features, such as main pulmonary artery diameter (MPAd), RV hypertrophy, RV dilation, and ventricular interdependence, have been strongly associated with PAH severity and prognosis.^9^^,^^10^^,^^11^ Importantly, CTPA offers several advantages over RHC, including its non-invasive nature, widespread availability, and ability to provide comprehensive structural and functional insights in a single examination. Emerging evidence suggests that CTPA-derived parameters correlate closely with RHC-confirmed PAH severity, offering a promising alternative or adjunct to invasive diagnostic procedures.^12^ By integrating these imaging metrics, clinicians can potentially reduce reliance on RHC, streamline diagnostic workflows, and improve patient outcomes.

While previous studies have highlighted individual CTPA-derived parameters associated with PAH, there is a lack of comprehensive, integrated models that leverage multiple imaging features to predict PAH probability and severity. The objective of this study is to identify CTPA-derived parameters associated with RHC-confirmed PAH and to develop a robust predictive model and nomogram for estimating the probability of PAH. By leveraging readily available CTPA imaging data, we aim to create a clinically accessible tool that enhances non-invasive risk stratification, bridges the diagnostic gap between symptom onset and RHC, and supports precision medicine strategies for PAH management. This approach has the potential to facilitate earlier diagnosis, reduce the need for invasive procedures, and improve patient outcomes.

## Results

### Patient characteristics

A total of 301 patients were included, with 203 in the training cohort and 98 in the validation cohort (Table 1 and Figure 1). In the training cohort, 80 patients (39.4%) had PAH and 123 (60.6%) did not; the validation cohort included 48 PAH patients (49.0%) and 50 non-pulmonary hypertension (non-PH) patients (51.0%). Significant baseline differences existed between PAH and non-PH groups in both cohorts.

**Table 1:** Baseline characteristics of the training and validation cohorts

Characteristics	Training (*n* = 203)			Validation (*n* = 98)		
	PAH (*n* = 80)	Non-PH (*n* = 123)	*p* value	PAH (*n* = 48)	Non-PH (*n* = 50)	*p* value
Age, years	48.6 ± 14.7	64.6 ± 15.2	<0.001	44.1 ± 15.6	64.2 ± 14.6	<0.001
Male, *n* (%)	13 (16.2%)	53 (43.1%)	<0.001	15 (31.2%)	18 (36.0%)	0.777
MPAd, mm	35.8 [33.1, 41.0]	28.1 [25.2, 31.4]	<0.001	41.0 [36.2, 45.9]	27.0 [25.0, 31.7]	<0.001
MPAa, cm^2^	11.1 [9.6, 14.5]	7.6 [6.1, 8.8]	<0.001	14.2 [11.6, 18.1]	7.5 [5.9, 8.4]	<0.001
LPAd, mm	23.0 [21.2, 26.7]	21.1 [19.3, 23.5]	<0.001	26.5 [23.1, 28.5]	21.0 [19.2, 23.6]	<0.001
LPAa, cm^2^	4.4 [3.7, 5.7]	3.7 [3.2, 4.5]	<0.001	5.4 [4.1, 6.6]	3.6 [3.0, 4.4]	<0.001
RPAd, mm	26.0 [23.0, 29.0]	23.0 [20.7, 26.4]	<0.001	29.2 [25.2, 31.9]	22.6 [20.6, 26.6]	<0.001
RPAa, cm^2^	5.5 [4.5, 6.8]	4.3 [3.5, 5.5]	<0.001	7.1 [5.3, 8.6]	4.0 [3.2, 5.5]	<0.001
AAd, mm	30.2 ± 5.3	34.6 ± 5.0	<0.001	28.7 ± 5.3	33.9 ± 4.6	<0.001
AAa, cm^2^	7.2 [5.9, 9.2]	9.2 [7.7, 11.0]	<0.001	6.5 [5.1, 8.2]	8.8 [7.6, 11.0]	<0.001
LVtd, mm	36.7 ± 7.8	41.5 ± 9.0	<0.001	37.6 ± 10.8	41.0 ± 9.3	0.094
LVld, mm	56.6 [50.8, 60.9]	62.1 [55.7, 68.9]	<0.001	56.8 [51.0, 62.6]	59.8 [54.0, 66.3]	0.157
LVa, cm^2^	17.2 [13.5, 20.8]	23.5 [17.8, 28.0]	<0.001	17.5 [13.5, 22.9]	23.1 [16.9, 25.7]	0.011
RVtd, mm	43.0 [35.8, 47.8]	36.4 [31.3, 40.2]	<0.001	43.7 [38.1, 51.8]	35.2 [29.6, 40.0]	<0.001
RVld, mm	63.2 [57.4, 70.9]	54.5 [49.7, 62.7]	<0.001	63.8 [58.9, 75.6]	54.8 [49.0, 62.5]	<0.001
RVa, cm^2^	20.5 [14.9, 25.0]	15.1 [11.6, 18.1]	<0.001	23.8 [17.4, 30.3]	14.9 [11.0, 18.1]	<0.001
LAap, mm	27.0 [22.3, 31.9]	30.2 [25.4, 33.5]	0.017	27.4 [24.2, 34.3]	30.8 [27.4, 34.4]	0.151
LAlr, mm	36.8 [30.3, 47.9]	40.3 [35.0, 46.5]	0.041	36.9 [32.8, 43.7]	41.2 [36.1, 48.1]	0.005
LAa, cm^2^	8.7 [5.9, 12.1]	9.5 [7.6, 12.2]	0.025	8.8 [6.9, 11.7]	9.8 [8.0, 12.4]	0.087
RAtd, mm	57.1 [50.3, 67.0]	46.5 [40.8, 51.5]	<0.001	61.8 [51.6, 69.7]	48.1 [40.5, 52.4]	<0.001
RAld, mm	50.6 [42.6, 59.2]	38.7 [34.0, 45.3]	<0.001	50.7 [42.4, 57.8]	39.4 [34.6, 46.0]	<0.001
RAa, cm^2^	25.6 [19.4, 33.8]	15.9 [12.4, 19.5]	<0.001	27.3 [20.8, 34.3]	16.5 [12.6, 20.1]	<0.001
Cobb	60.9 [50.7, 71.9]	42.2 [36.2, 48.4]	<0.001	57.6 [46.0, 65.8]	42.1 [37.1, 47.7]	<0.001
IVSA	156.6 [144.7, 165.7]	119.3 [109.9, 127.6]	<0.001	156.6 [142.5, 165.9]	121.9 [111.2, 127.8]	<0.001
RVAWT, mm	4.9 [4.2, 5.6]	3.9 [3.4, 4.4]	<0.001	5.9 [4.9, 8.4]	3.8 [3.4, 4.3]	<0.001
IVST, mm	9.4 [7.9, 10.4]	10.3 [8.7, 11.7]	0.001	8.8 [7.9, 11.6]	10.0 [8.8, 11.6]	0.087
RVOTd, mm	26.8 ± 5.4	24.2 ± 4.2	<0.001	27.4 ± 4.7	24.1 ± 4.1	<0.001
RVOTW, mm	4.9 [4.3, 6.0]	3.7 [3.3, 4.2]	<0.001	5.8 [5.0, 7.5]	3.5 [3.2, 4.2]	<0.001
Reflux, *n* (%)	–	–	<0.001	–	–	<0.001
None	15 (18.8%)	87 (70.7%)	–	10 (20.8%)	39 (78.0%)	–
Mild	19 (23.8%)	30 (24.4%)	–	21 (43.8%)	10 (20.0%)	–
Moderate	29 (36.2%)	5 (4.1%)	–	7 (14.6%)	1 (2.0%)	–
Severe	17 (21.2%)	1 (0.8%)	–	10 (20.8%)	0 (0.0%)	–
Flat, *n* (%)	50 (62.5%)	5 (4.1%)	<0.001	33 (68.8%)	1 (2.0%)	<0.001

Continuous variables are expressed as mean ± SD or as median (interquartile range), and categorial variables are expressed as number (%).

MPAd, main pulmonary artery diameter (mm); MPAa, main pulmonary artery area (cm^2^); LPAd, left pulmonary artery diameter (mm); LPAa, left pulmonary artery area (cm^2^); RPAd, right pulmonary artery diameter (mm); RPAa, right pulmonary artery area (cm^2^); AAd, aortic arch diameter (mm); AAa, aortic arch area (cm^2^); LVtd, left ventricle transverse diameter (mm); LVld, left ventricle longitudinal diameter (mm); LVa, left ventricle area (cm^2^); RVtd, right ventricle transverse diameter (mm); RVld, right ventricle longitudinal diameter (mm); RVa, right ventricle area (cm^2^); LAap, left atrium anteroposterior diameter (mm); LAlr, left atrium laterolateral diameter (mm); LAa, left atrium area (cm^2^); RAtd, right atrium transverse diameter (mm); RAld, right atrium longitudinal diameter (mm); RAa, right atrium area (cm^2^); Cobb, Cobb angle; IVSA, interventricular septal angle; RVAWT, right ventricular anterior wall thickness (mm); IVST, interventricular septal thickness (mm); RVOTd, right ventricular outflow tract diameter (mm); RVOTW, right ventricular outflow tract wall thickness (mm); reflux, *n* (%), reflux severity (none, mild, moderate, and severe); flat, *n* (%), flat morphology prevalence .

PAH patients were notably younger (training: 48.6 ± 14.7 vs. 64.6 ± 15.2 years, *p* < 0.001; validation: 44.1 ± 15.6 vs. 64.2 ± 14.6 years, *p* < 0.001) and had a lower male proportion in the training cohort (16.2% vs. 43.1%, *p* < 0.001), though gender differences were not significant in the validation cohort (31.2% vs. 36.0%, *p* = 0.777).

### CTPA-derived parameters

PAH patients exhibited significantly larger pulmonary artery measurements in both cohorts. In the training cohort, MPAd: 35.8 vs. 28.1 mm, *p* < 0.001) and cross-sectional area (MPA area [MPAa]: 11.1 vs. 7.6 cm^2^, *p* < 0.001), as well as right pulmonary artery diameter (RPAd: 26.0 vs. 23.0 mm, *p* < 0.001) and area (RPAa: 5.5 vs. 4.3 cm^2^, *p* < 0.001), were greater in PAH cases. Identical trends were observed in the validation cohort (MPAd: 41.0 vs. 27.0 mm, *p* < 0.001; MPAa: 14.2 vs. 7.5 cm^2^, *p* < 0.001; RPAd: 29.2 vs. 22.6 mm, *p* < 0.001; RPAa: 7.1 vs. 4.0 cm^2^, *p* < 0.001).

RV dimensions and wall thickness were also significantly greater in PAH patients. Training cohort data showed larger RV transverse diameter (RVtd: 43.0 vs. 36.4 mm, *p* < 0.001) and anterior wall thickness (RVAWT: 4.9 vs. 3.9 mm, *p* < 0.001), replicated in the validation cohort (RVtd: 43.7 vs. 35.2 mm, *p* < 0.001; RVAWT: 5.9 vs. 3.8 mm, *p* < 0.001). These findings highlight RV remodeling as a key correlation of PAH.

### Parameter selection

To identify the most relevant predictors of PAH, logistic regression in the training cohort identified three independent predictors of PAH (Table 2): interventricular septal angle (IVSA; odds ratio [OR]: 1.123, *p* < 0.001), interventricular septal thickness (IVST; OR: 0.683, *p* = 0.048), and RV outflow tract wall thickness (RVOTW; OR: 2.133, *p* = 0.005). The events per variable (EPV), defined as the number of events divided by the number of variables, was approximately 26.7, exceeding the empirical threshold of 10. This indicates that the model is relatively stable with a low risk of overfitting. Also, the least absolute shrinkage and selection operator (LASSO) also demonstrated these variables associated with resting mPAP >20 mmHg (Figure S2), forming the basis for subsequent analysis.

**Table 2:** Multivariable logistic regression analysis for PAH prediction

Variable	β	SE	Z	P	OR (95% CI)
Age	−1.235	0.735	−1.68	0.093	0.291 (0.069–1.228)
MPAd	0.123	0.068	1.80	0.072	1.130 (0.989–1.292)
LVa	−0.071	0.071	−1.00	0.319	0.932 (0.811–1.071)
IVSA	0.116	0.034	3.46	<0.001	1.123 (1.052–1.199)
IVST	−0.381	0.193	−1.98	0.048	0.683 (0.469–0.997)
RVOTW	0.757	0.268	2.83	0.005	2.133 (1.262–3.606)
Reflux					
Mild	−1.050	1.061	−0.99	0.322	0.350 (0.044–2.800)
Moderate	2.209	1.045	2.11	0.035	9.106 (1.175–70.550)
Severe	3.803	1.564	2.43	0.015	44.822 (2.091–960.782)

Age was divided into ≥60 and <60 years. MPAd, main pulmonary artery diameter (mm); LVa, left ventricle area (cm^2^); IVSA, interventricular septal angle; IVST, interventricular septal thickness (mm); RVOTW, right ventricular outflow tract wall thickness (mm).

### Model performance and nomogram

The nomogram, constructed using the independent predictors by both logistic and LASSO regression models, provided a user-friendly tool for estimating PAH probability (Figure 2).

**Figure 1:**
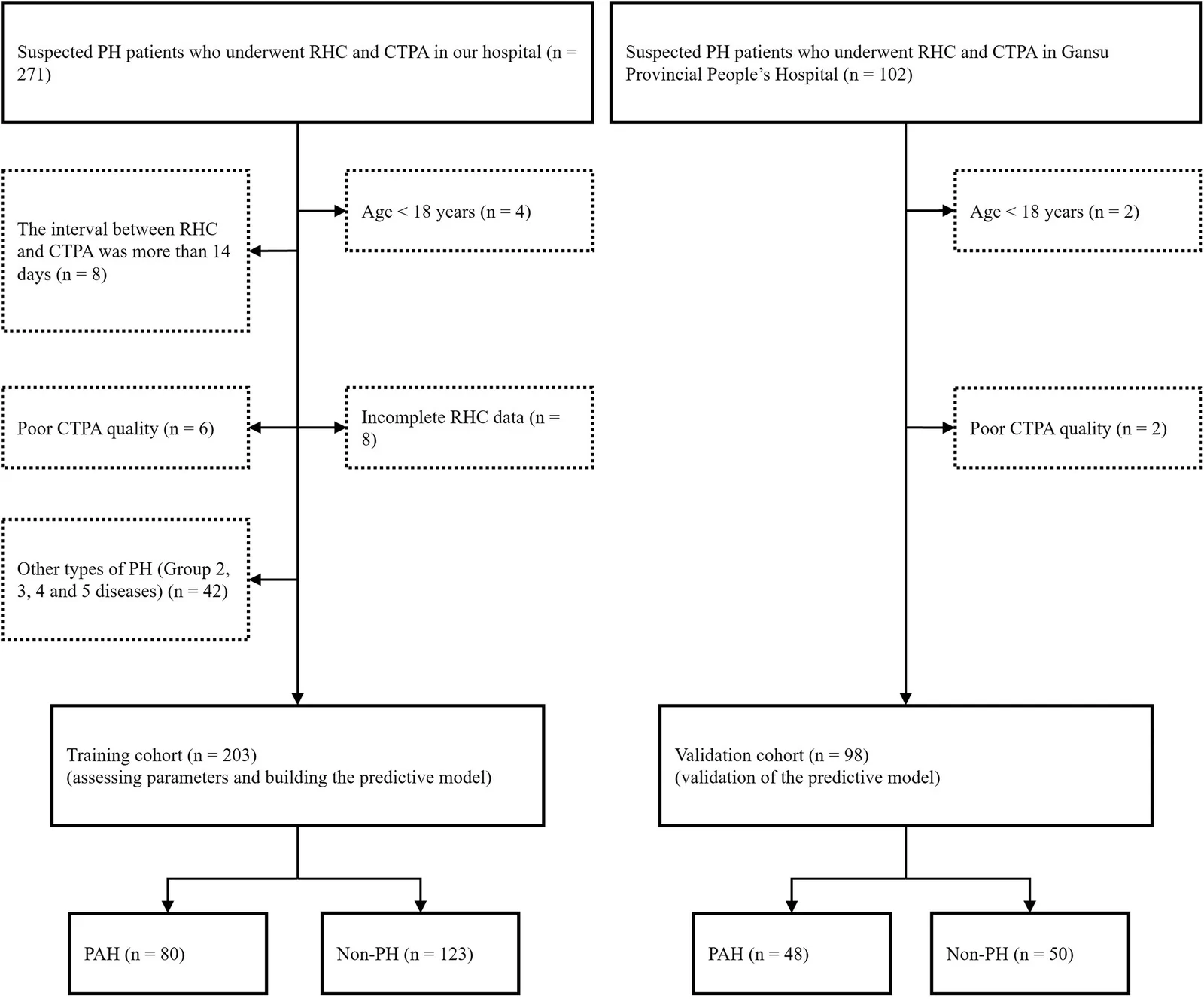
Study flowchart

**Figure 2:**
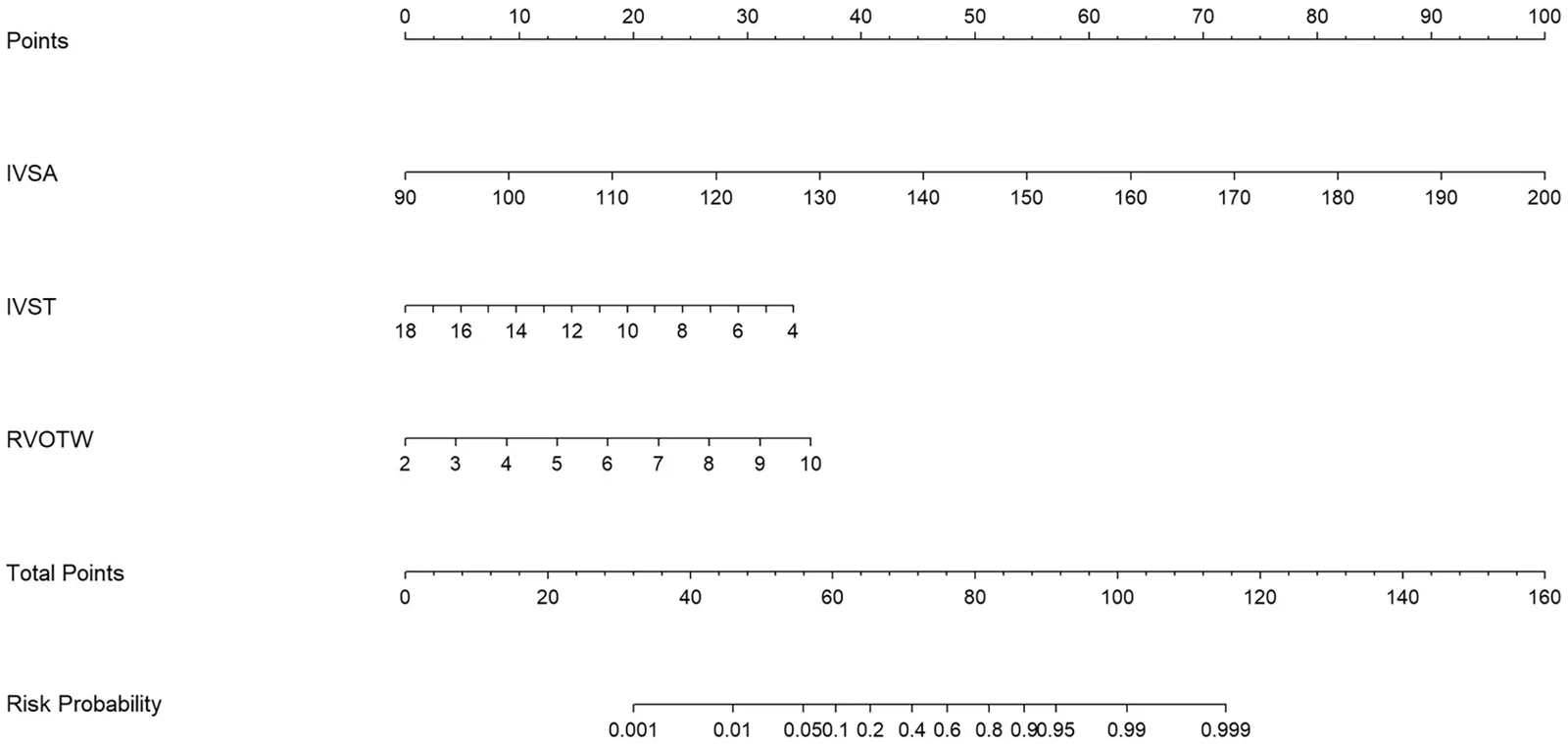
Nomogram for predicting PAH probability The nomogram incorporates IVSA, IVST, and RVOTW to estimate the probability of PAH. Individual scores are derived by projecting each predictor upward to the top 'Points' scale. The total score is then calculated and projected downward to the 'Risk Probability' scale to determine the corresponding probability of PAH occurrence. IVSA, interventricular septal angle; IVST, interventricular septal thickness (mm); RVOTW, right ventricular outflow tract wall thickness (mm).

The predictive model demonstrated strong discriminatory ability in both the training and validation cohorts, with an area under the receiver operating characteristic (ROC) curve (AUC) of 0.964 (95% confidence interval [CI]: 0.929–0.985) in the training cohort and 0.969 (95% CI: 0.912–0.993) in the validation cohort (Figure 3).

**Figure 3:**
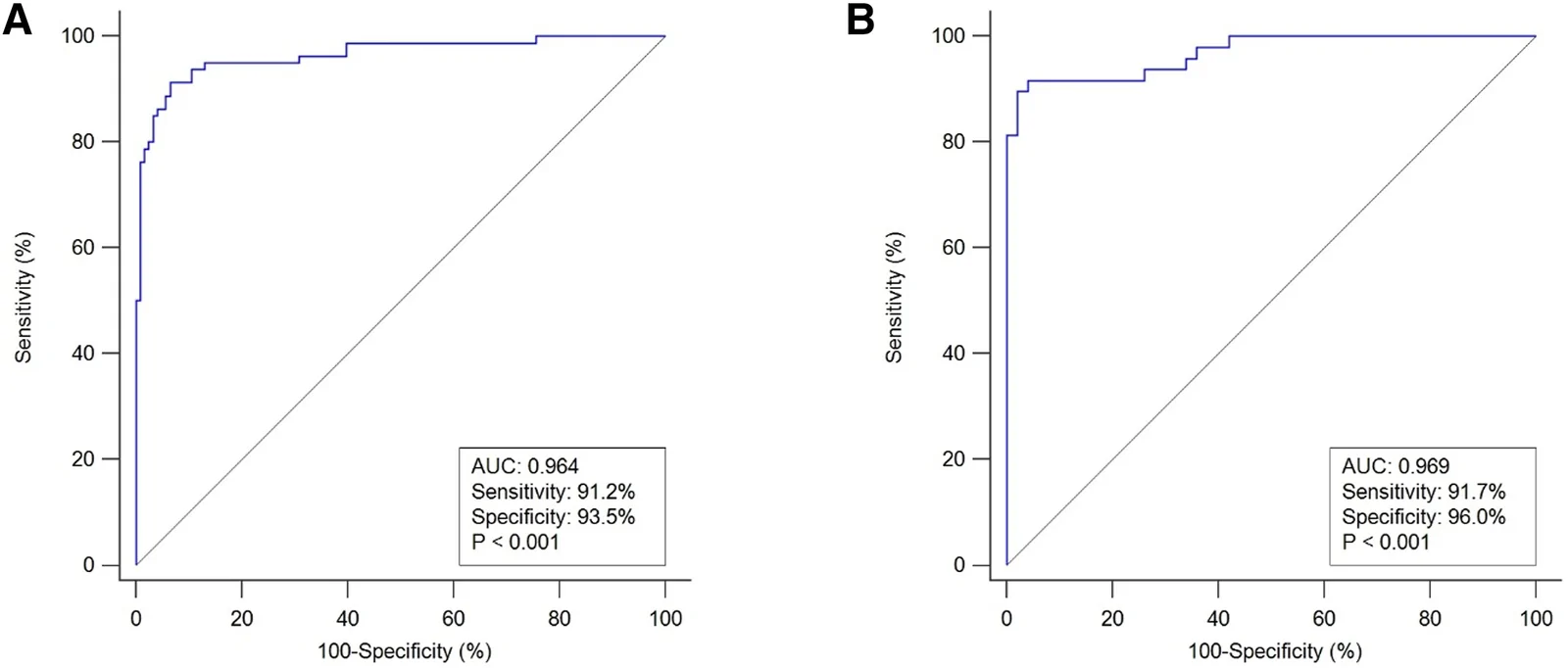
ROC curves for the predictive model (A and B) Receiver operating characteristic (ROC) curves demonstrating the model’s discriminatory ability in the training cohort (A) and validation cohort (B).

### Internal validation via bootstrap resampling

The bootstrap-corrected AUC was 0.960 (95% CI: 0.938–0.991), representing a minimal decrease from the apparent AUC of 0.964 (optimism = 0.004). The shrinkage coefficient of 0.993 indicated that the regression coefficients required negligible adjustment. Calibration statistics were favorable: the intercept was 0.000 (95% CI: −0.603–0.541) and slope was 1.000 (95% CI: 0.756–1.560). The Brier score was 0.062 (95% CI: 0.040–0.086). These results confirm that the model’s high discrimination ability is genuine with minimal overfitting risk (Table S1).

### Calibration capability of the optimal model

The calibration capability of the optimal model was assessed using a calibration curve (Figure 4). The curve illustrates the agreement between the predicted probabilities of PAH and the observed outcomes. The model’s predictions closely aligned with the 45-degree reference line, indicating excellent calibration. This suggests that the model accurately estimates the probability of PAH across different risk thresholds, making it reliable for clinical application.

**Figure 4:**
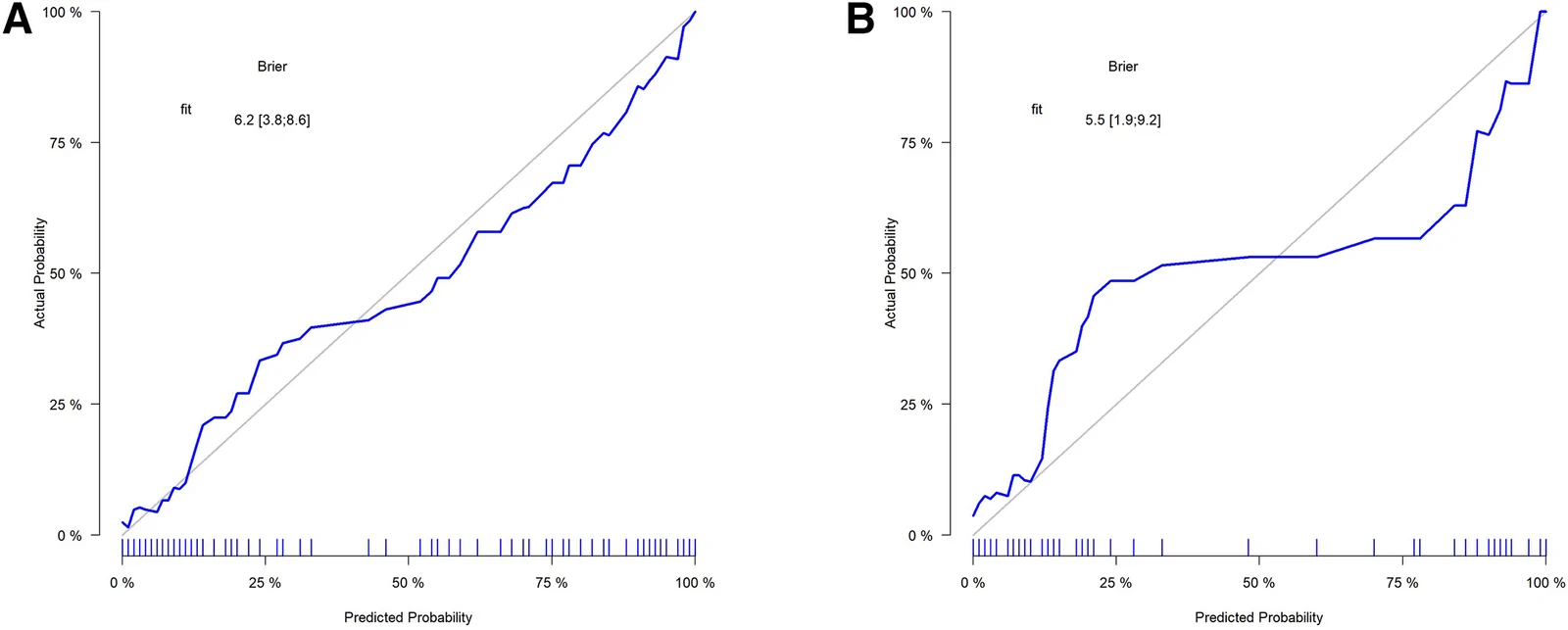
Calibration curve of the predictive model (A and B) The *x* axis represents the predicted probability of PAH, and the *y* axis represents the observed probability. The solid line represents the model’s calibration, while the dashed line represents the ideal calibration (45-degree reference line).

### Clinical effectiveness of the nomogram

The clinical effectiveness of the nomogram was evaluated using decision curve analysis (DCA) (Figure 5). Results indicate that the nomogram is clinically effective and can assist clinicians in making informed decisions by optimizing the balance between true positives and false positives.

**Figure 5:**
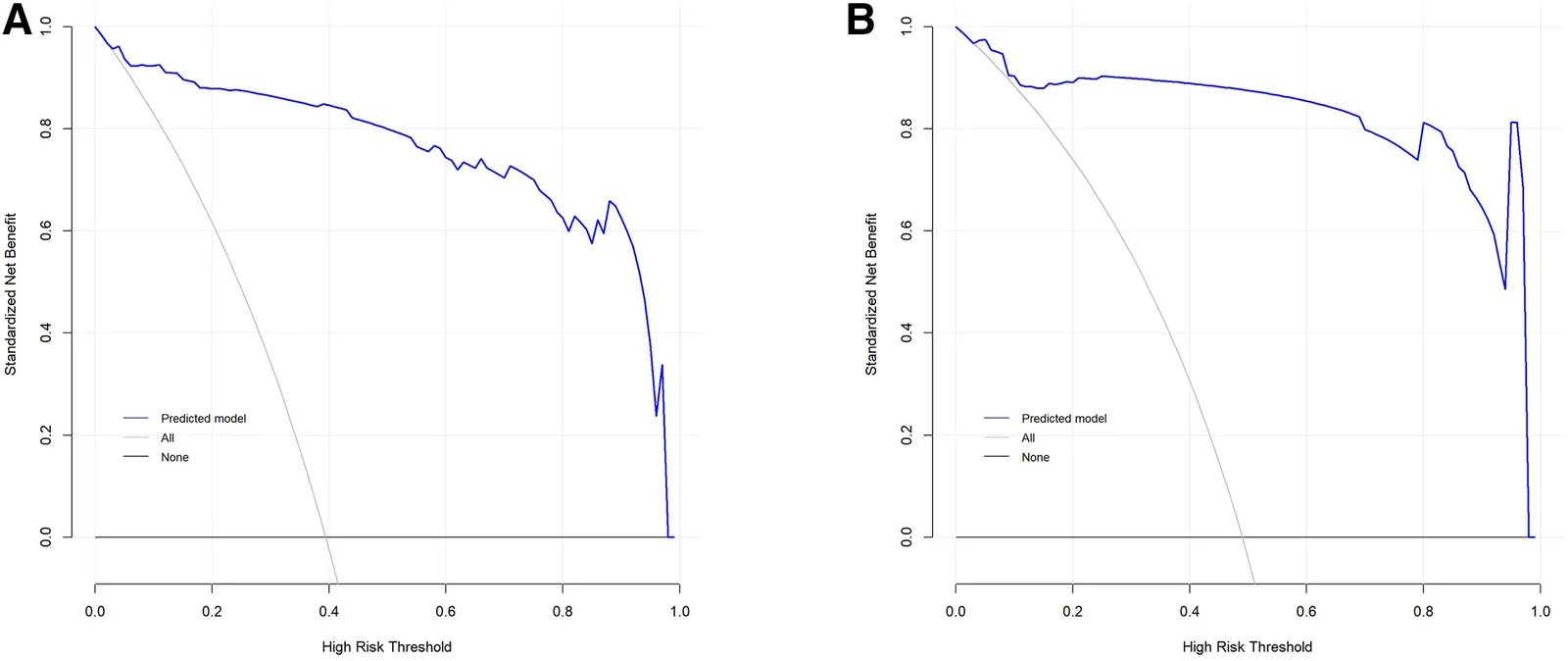
Decision curve analysis of the nomogram (A and B) The *x* axis represents the threshold probability for PAH diagnosis, and the *y* axis represents the net benefit. The solid line represents the net benefit of using the nomogram, while the dashed and horizontal lines represent the net benefit of diagnosing all or no patients as having PAH, respectively.

### Comparative models performance analysis

The ROC analysis demonstrated that model 4 (combining IVSA, IVST, and RVOTW) achieved the highest diagnostic accuracy in both the training cohort (AUC = 0.964, 95% CI: 0.929–0.985) and validation cohort (AUC = 0.969, 95% CI: 0.912–0.993), with sensitivity >90% and specificity >93%. Model 1 (IVSA alone) also showed excellent discrimination with AUCs of 0.950 in the training cohort and 0.955 in the validation cohort, while model 2 (IVST alone) performed poorly with AUCs of 0.642 (training cohort) and 0.600 (validation cohort). Model 3 (RVOTW alone) exhibited moderate performance with training and validation AUCs of 0.808 and 0.924, respectively. Statistical comparisons confirmed that model 4 significantly outperformed model 2 and 3 (all *p* < 0.05), but not model 1 (*p* > 0.05) (Figure S3 and Table S2).

DCA revealed that model 4 provided the highest net benefit across a wide range of threshold probabilities in both cohorts, outperforming models 1–3 and the extreme assumptions (all/no PAH). Model 1 also showed clinical utility but was inferior to model 4. Model 2 had minimal net benefit, aligning with its low AUC. The results support the clinical utility of model 4 in the diagnosis of PAH (Figure S4).

The integrative analysis of ROC and DCA underscores that model 4 (including IVSA, IVST, and RVOTW) is the most robust diagnostic tool for PAH, balancing strong discriminative power (AUC >0.96) and clinical applicability. While model 1 (IVSA alone) may serve as a feasible alternative, the suboptimal performance of model 2 (IVST alone) suggests limited independent utility.

### Subgroup analysis

To evaluate the robustness of the predictive model across distinct demographic subgroups, we performed subgroup analyses within the validation cohort (*n* = 98). As depicted in Table S3, the model retained excellent discriminatory efficacy across all subgroups. Age-stratified analysis demonstrated an AUC of 0.981 (95% CI: 0.952–1.000) for patients <60 years and 0.877 (95% CI: 0.689–1.000) for those ≥60 years; no statistically significant intergroup difference was observed (DeLong test: *p* = 0.296). Similarly, gender-stratified analysis yielded an AUC of 0.963 (95% CI: 0.900–1.000) for males and 0.955 (95% CI: 0.891–1.000) for females, with no significant intergroup difference (*p* = 0.854). These findings indicate that the predictive efficacy of the model is consistent across demographic subgroups and is primarily driven by pathophysiological imaging parameters rather than demographic characteristics.

### Sensitivity analysis

To assess the robustness of the predictive model, sensitivity analyses were performed by incorporating two clinically relevant parameters—MPAd and the ratio of MPAd to aortic arch diameter (rPA)—into the baseline model (model 1).

All variance inflation factor (VIF) values for each model were below 5, indicating no serve multicollinearity concerns (Table S4).

Model performance was systematically assessed across three key domains. Discrimination: neither model 2 (incorporating MPAd) nor model 3 (incorporating rPA) demonstrated a statistically significant improvement in the AUC values compared with model 1 in either the training or validation cohort (all *p* > 0.05) (Figure S5 and Table S5). Calibration and overall predictive accuracy: calibration curve analysis and quantitative metrics indicated that model 1 achieved excellent agreement between predicted probability and actual proportion. In the validation cohort, model 1 exhibited the calibration slope closest to the ideal value of 1.000 (0.946, 95% CI: 0.895–0.997) and had the low Brier score (0.055) reflecting superior overall predictive accuracy (Figure S6 and Table S6). Clinical utility: DCA showed comparable clinical net benefit across all models. Model 2 and 3 provided modest additional net benefit within specific threshold probability ranges, without demonstrating consistent superiority (Figure S7 and Table S7).

In summary, the inclusion of MPAd or rPA did not yeild significant incremental value in terms of discrimination, calibration, or clinical utility. Model 1 remained the most parsimonious and well-calibrated model.

## Discussion

This study demonstrates that CTPA-derived structural parameters—specifically pulmonary artery dimensions, cross-sectional areas, and RV metrics—are strongly associated with RHC-confirmed PAH. Multivariable logistic and LASSO regression analyses identified three primary predictors: IVSA, IVST, and RVOTW. The resulting predictive model demonstrated high diagnostic robustness and clinical utility, offering a rapid, non-invasive approach to preliminary risk assessment and diagnostic triage.

PAH is pathologically defined by progressive vascular and RV remodeling, both of which are readily captured by CTPA.^13^^,^^14^ The significant differences observed in pulmonary artery dimensions (e.g., MPAd and RPAd) and RV measurements (e.g., RVtd and RVAWT) between PAH and non-PH groups highlight the role of these parameters in reflecting the hemodynamic and morphological changes associated with PAH.^15^^,^^16^ Specifically, arterial dilation and RV wall thickening reflect compensatory responses to chronic RV pressure overload.^17^ Given the rising global burden of PAH,^18^^,^^19^^,^^20^ these findings confirm the value of standardized CTPA metrics as reliable visual markers of pulmonary circulatory remodeling.

The incorporation of IVSA, IVST, and RVOTW into our model reflects distinct physiological dimensions of RV adaptation to elevated afterload. The interventricular septum is central to cardiac mechanics under pressure overload.^21^ In PAH, the RV faces elevated pulmonary artery pressures, leading to compensatory mechanisms such as hypertrophy and remodeling of the interventricular septum. Increased IVST reflects these adaptive long-term changes and is a direct indicator of the RV’s response to chronic pressure overload.^22^^,^^23^ Meanwhile, IVSA captures overall morphological remodeling driven by disease progression.^24^ Similarly, RVOTW is a marker of RV hypertrophy, which develops as a consequence of chronic pressure overload and is essential for distinguishing PAH from non-PH cases.^25^^,^^26^

Together, these parameters are more directly associated with the pathophysiological mechanisms of PAH, capturing both the morphological and functional adaptations of the RV to pressure overload. By combining these parameters, we can achieve a more holistic understanding of the disease state of PAH.^27^^,^^28^ Intriguingly, IVST was consistently thinner in PAH patients than in non-PH controls across both cohorts (training: 9.4 vs. 10.3 mm, *p* = 0.001; validation: 8.8 vs. 10.0 mm, *p* = 0.087), yielding a paradoxical negative association in multivariable regression (OR < 1). We propose three potential explanations for this counterintuitive finding. First, sustained RV pressure overload causes leftward septal displacement, compromising left ventricular (LV) filling and inducing downstream LV myocardial disuse atrophy and fibrosis.^23^ Thus, when adjusted for IVSA (septal geometry) and RVOTW (RV hypertrophy), IVST primarily captures LV-side thinning rather than classic septal hypertrophy. Secondly, older age and a higher male proportion in the non-PH group may predispose to a higher prevalence of hypertension or ischemic LV remodeling, thereby increasing the baseline IVST in the control group. Thirdly, IVST measurements were obtained at the four-chamber view level on CTPA. In PAH patients, the characteristic D-shaped distortion of the LV and torsional deformation of the interventricular septum may lead to underestimation of linear septal thickness due to oblique measurement planes.^3^^,^^10^^,^^22^ These chosen predictors—IVSA, IVST, and RVOTW—provide a more nuanced assessment of the RV’s response to increased pulmonary vascular resistance.

Although electrocardiography (ECG) is widely accessible and routinely used for initial PAH screening (typically indicating right axis deviation and RV hypertrophy), it primarily reflects electrophysiological changes. Also, its sensitivity is volatile and it cannot directly quantify structural changes. In line with current clinical guidelines, transthoracic echocardiography (TTE) remains the cornerstone of non-invasive screening for PAH. However, its capacity for precise structural quantification is limited by suboptimal acoustic windows and significant operator dependence.

In contrast, CTPA provides high-resolution, reproducible anatomical data of the pulmonary vasculature and cardiac chambers in a single acquisition. Given its routine use in excluding pulmonary embolism and evaluating unexplained dyspnea, the integration of CTPA-derived parameters into clinical assessment imposes minimal incremental burden. The predictive model enables rapid estimation of quantitative PAH probability using pre-existing CTPA images under specific clinical scenarios, including non-specific ECG findings, inadequate TTE windows, unmeasurable tricuspid regurgitation velocity (TRV), or where RHC access is restricted. Importantly, this study does not position CTPA as a replacement for the conventional diagnostic pathway initiated by ECG and TTE. Rather, it aims to serve as a complementary tool to improve the diagnostic efficiency and support risk-stratified referral decisions in the evaluation of suspected PAH (Figure S8).

Artificial intelligence (AI)-based automated volumetric measurements are a promising tool for assessing PAH using CTPA. Studies have demonstrated that main pulmonary artery volume, measured by three-dimensional (3D) volumetric analysis, strongly correlates with mPAP (r = 0.74–0.76) and yields an AUC of 0.90–0.934 for PAH diagnosis, indicating excellent diagnostic performance.^6^^,^^29^ Similarly, automated left atrial volume effectively distinguishes left heart disease associated with PH (LHD-PH).^30^^,^^31^ AI-derived automated segmentation and volumetric analysis offer high reproducibility, time efficiency, and the capacity to extract complex 3D imaging features.^6^^,^^29^^,^^30^^,^^32^ However, wide clinical deployment of AI remains hindered by algorithm opacity, vendor lock-in, and lack of prospective validation in resource-limited settings^33^^,^^34^; its clinical translation requires validation in large-scale prospective cohorts, and the decision-making processes of existing algorithms often lack explicit anatomical interpretability, compromising result transparency and clinical trust.^35^^,^^36^ In this context, the model developed in this study, which relies on manual measurements, offers immediate clinical accessibility and operational transparency, serving as an interpretable baseline for future automated pipelines. We recognize, however, that manual measurements have inherent limitations, including prolonged time investment and inter-observer variability. As advanced AI algorithms evolve, these key parameters are poised to transition toward an efficient, standardized, and interpretable intelligent diagnostic framework, built upon precise, standardized measurements. The integration of CTPA-derived parameters into predictive models can facilitate timely intervention, improve risk stratification, and guide therapeutic decision-making, particularly in resource-limited settings where RHC may not be readily accessible.

Finally, although sensitivity analyses indicated that adding classic parameters (MPAd and rPA) yielded no significant AUC gain over our core model, these vascular metrics retain clear clinical relevance.^37^^,^^38^ Combining vascular metrics (MPAd/rPA) with ventricular adaptive parameters (IVSA/IVST/RVOTW) may further refine future risk models. Future studies should aim to harness the collective power of these parameters to optimize predictive models and inform clinical decision-making in the management of this complex disease.

The findings of this study open several promising avenues for future research and clinical application. First, our model and nomogram warrant external validation across diverse multi-center cohorts and distinct PAH etiologies. Second, integrating advanced imaging techniques, such as 4D flow MRI or dual-energy CT, could offer deeper hemodynamic insights, further enhancing model accuracy. Third, incorporating machine learning algorithms or AI may improve the predictive power by identifying complex patterns and interactions among predictors. Finally, longitudinal studies are needed to assess the utility of CTPA indices in predicting disease progression and treatment response, thereby solidifying their role in clinical practice.

In conclusion, this study highlights the utility of CTPA-derived parameters in identifying PAH and developing a reliable predictive model. The nomogram offers a practical, non-invasive tool for clinicians, with significant potential to improve early diagnosis, risk stratification, and clinical decision-making in PAH. Future research should focus on validating and refining the model, integrating advanced imaging techniques, and exploring its application in diverse clinical settings.

### Limitations of the study

Despite its strengths, this study has several limitations. First, the retrospective design may introduce selection bias, and the findings need to be validated in prospective, multi-center studies to ensure generalizability. Second, the sample size, although adequate for model development, may limit the detection of less common predictors or subtle associations. Third, the external validity of our model is limited by both demographic/technical homogeneity and single-observer measurement. All data were derived from tertiary hospitals in China using predominantly single-vendor CT scanners, which may restrict applicability to other healthcare settings, imaging platforms, or populations. Additionally, while single-observer assessment ensured internal consistency, it does not reflect measurement variability in real-world multi-reader clinical practice. Future multi-center, multi-vendor, and multi-ethnic validation studies incorporating multiple observers are needed to establish model’s generalizability and clinical robustness. Finally, while the model demonstrates strong discriminatory ability, its performance in specific PH subtypes (e.g., PAH vs. chronic thromboembolic PH) requires further investigation. Future studies should also explore the integration of additional biomarkers or clinical parameters to enhance the model’s predictive accuracy.

## Resource availability

### Lead contact

Requests for further information, resources, and reagents should be directed to and will be fulfilled by the lead contract, Prof. Yanli Zhou (zhyl88@qq.com).

### Materials availability

This study did not generate new unique reagents.

### Data and code availability

• The patient data reported in this study cannot be deposited in a public repository because of patient privacy and confidentiality. To submit a request for potential access, contact the lead contact.
• This paper does not report original code.
• Any additional information required to reanalyze the data reported in this paper is available from the lead contact upon reasonable request.

## Acknowledgments

This study was supported by the State Key Laboratory for Innovation and Transformation of Luobing Theory, the General Program of the 10.13039/501100001809National Natural Science Foundation of China (grant nos. 82370389 and 81970339 to X.L.; 82400458 to S.L.), the National High Technology Research and Development Program of China (grant no. 2017YFC1700505 to X.L.), and Nanjing Postdoctoral Research Support Program (2025BSH073).

## Author contributions

Conceptualization, H.Y., I.C., G.L., Y. Xu., and Y.Z.; methodology, H.Y., I.C., and Y.C.; software, X.Z. and S.L.; formal analysis, H.Y., I.C., and Y. Xing.; investigation, H.Y., X.Z., and W.L.; data curation, G.L. and Y. Xing.; validation, G.L., Y. Xing., and J.H.; resources, G.L., W.L., and J.H.; writing – original draft, H.Y. and I.C.; writing – review and editing, J.H., X.L., Y. Xu., and Y.Z.; visualization, X.Z., J.H., and Y.C.; supervision, S.L., Y. Xu., and Y.Z.; funding acquisition, S.L., X.L., Y. Xu., and Y.Z. All authors had read and commented on the manuscript, gave final approval, and agreed to be accountable for all aspects of work ensuring integrity and accuracy.

## Declaration of interests

The authors declare no competing interests.

## STAR★Methods

### Key resources table

**Table:** 

REAGENT or RESOURCE	SOURCE	IDENTIFIER
**Software and algorithms**		

SPSS software (version 25.0)	IBM SPSS software	https://www.ibm.com/products/spss
R software (version 4.0.0)	The R Foundation	https://www.r-project.org/foundation/
Syngo.via (version VB40B_HF08)	Siemens Healthcare GmbH, Erlangen, Germany	https://www.siemens-healthineers.com/syngo.via; GTIN: 04056869237633

### Experimental model and study participant details

#### Study design

This was a retrospective, multicenter study designed to develop and validate a predictive model and nomogram for identifying PAH using CTPA parameters. The study included a training cohort for model development and an independent validation cohort to assess the model’s generalizability and performance, with PAH defined by the gold-standard RHC criteria.

#### Ethics approval and consent to participant

Study protocol was approval by the independent institutional ethical committee of the First Affiliated Hospital with Nanjing Medical University and conducted in accordance with the Declaration of Helsinki (Approval No. 2022-SR-506).

#### Participants

The training cohort included patients suspected of having pulmonary hypertension (PH) from the First Affiliated Hospital with Nanjing Medical University between May 2021 and December 2024. The validation cohort comprised patients with clinically suspected PH managed at Gansu Provincial People’s Hospital and Sichuan Provincial People’s Hospital during the same time frame. All participants underwent both CTPA and RHC within 14 days (to ensure hemodynamic stability), with PAH confirmed by RHC according to the Sixth World Symposium on Pulmonary Hypertension: mPAP >20 mmHg, PCWP ≤15 mmHg, and PVR >2 Wood units·m^2^ at rest.^3^

The exclusion criteria for the study were as follows: age younger than 18 years, incomplete RHC data, a time interval exceeding 14 days between CTPA and RHC, incomplete bilateral lung CTPA scans or suboptimal image quality, and other subtypes of PH (Group 2, 3, 4 and 5).

Patients were stratified into PAH and non-PH groups based on RHC findings. After screening, the training cohort included 80 PAH and 123 non-PH, with mean ages of 48.6 ± 14.7 and 64.6 ± 15.2 years, respectively. Male proportions were 16.2% in the PAH group and 43.1% in the non-PH group. The validation cohort consisted of 48 patients with PAH and 50 non-PH controls, with mean ages of 44.1 ± 15.6 years and 64.2 ± 14.6 years, respectively. Males accounted for 31.2% of the PAH group and 36.0% of the non-PH group (Figure 1 and Table 1).

#### Variables

Demographic, clinical, and imaging variables were collected, including age, gender, and reflux severity. CTPA-derived parameters were used as predictors, with PAH confirmed by RHC as the primary outcome.

All patients underwent RHC within 72 h of hospitalization. For hemodynamic assessment, a 7F Swan-Ganz catheter (Edwards Lifesciences, Irvine, CA) was inserted from right internal jugular venous approach and zeroed at the axillary midline level. Mean right atrial pressure, right ventricular pressure, systolic pulmonary artery pressure (sPAP), diastolic pulmonary artery pressure (dPAP), mPAP, PAWP were measured. Cardiac output (CO) was assessed in triplicate by thermodilution. PVR was calculated as (mPAP - PAWP)/CO.

### Method details

#### Imaging protocol

CTPA was performed using standardized protocols across both centers. Images were acquired using multidetector computed tomography (CT) scanners (Siemens Healthineers, Forchheim, Germany) with a slice thickness of 1 mm. Contrast enhancement was achieved using iodinated contrast media injected at a rate of 4–5 mL/s, followed by a saline flush. Image acquisition was timed to ensure optimal visualization of the pulmonary arteries.

#### Analysis of CTPA images and conventional data extraction

Phases with good image quality were manually selected for analysis. All CTPA measurements in this study were conducted on the same CT scanner (Siemens Healthineers, Forchheim, Germany) and dedicated post-processing workstation (Syngo.Via) to minimize systematic bias attributable to hardware or software variability. To reduce inter-observer variability, all images were independently analyzed offline by a senior cardiovascular radiologist with over five years of specialized experience in cardiovascular CT imaging, who remained blinded to patients’ clinical information and RHC results. For each patient, all included CTPA parameters were measured three times independently, and the mean value was calculated for inclusion in subsequent analyses to mitigate random measurement error. The following CTPA-derived parameters were extracted:

1. Pulmonary artery dimensions: main pulmonary artery diameter (MPAd), left pulmonary artery diameter (LPAd), and right pulmonary artery diameter (RPAd).
2. Cross-sectional areas: main pulmonary artery area (MPAa), left pulmonary artery area (LPAa), and right pulmonary artery area (RPAa).
3. Ventricular dimensions: left ventricular transverse diameter (LVtd), right ventricular transverse diameter (RVtd), and ventricular areas.
4. Wall thickness: right ventricular anterior wall thickness (RVAWT) and interventricular septal thickness (IVST).
5. Reflux: The severity of tricuspid regurgitation is simply classified into four grades based on the extent of contrast agent reflux into the inferior vena cava (IVC) or hepatic veins. These grades are defined as follows: none (no reflux into IVC), mild (reflux into the IVC but not into the hepatic veins), moderate (reflux into the proximal hepatic veins), and severe (reflux into the distal hepatic veins).

#### Development and validation

The predictive model was developed using the training cohort. Logistic regression analysis was employed to identify independent predictors of PAH. A nomogram was constructed to visualize the model and provide a user-friendly tool for estimating PAH probability. The model’s performance was evaluated using the validation cohort, assessing discrimination, calibration, and clinical utility.

### Quantification and statistical analysis

Continuous variables were analyzed using means and standard deviations (SDs) or medians and interquartile range, depending on the distribution of the data. Continuous variables were compared using t-tests or Mann-Whitney U tests, while categorical variables were analyzed using chi-square tests.

#### Model development and internal validation

The variable selection process in the training cohort (*n* = 203) was performed according to the following steps (Figure S1). Firstly, baseline demographic characteristics and CTPA-derived metrics were collected for patients who met the predefined inclusion and exclusion criteria. Secondly, candidate variables with a *p* < 0.1 in univariate analysis were selected for subsequent regularization modeling. Thirdly, Least Absolute Shrinkage and Selection Operator (LASSO) regression was conducted using 10-fold cross-validation to identify the optimal regularization parameter λ based on the one-standard error (1-SE) criterion, thereby selecting features demonstrating consistent predictive performance. Subsequently, collinearity among the selected variables was assessed using the generalized variance inflation factor (VIF), and any variable with a VIF >5 was removed to mitigate multicollinearity. Finally, the final predictive model was constructed through multivariate logistic regression, incorporating both statistical significance and clinical interpretability.

The area under the receiver operating characteristic curve (AUC) was calculated to assess discrimination. To mitigate overfitting risk and ensure model robustness, we implemented a comprehensive validation framework: (a) The events per variable (EPV) was calculated as a preliminary check for overfitting risk; (b) Variance inflation factor (VIF) was computed to assess multicollinearity among predictor variables; (c) Internal validation via bootstrap resampling (1,000 iterations) was conducted to estimate optimism-corrected performance metrics, including the optimism-corrected AUC, shrinkage coefficients, calibration statistics (intercept and slope), and Brier scores with 95% confidence interval (CI).

#### Model performance assessment

Model Performance was evaluated in three dimensions: (a) Discrimination: AUC values with 95% CIs were reported for both training and validation cohorts; (b) Calibration: Calibration curves were plotted. Calibration slopes and intercepts, and Brier scores were calculated with 95% CIs; (c) Clinical Utility: Decision curve analysis (DCA) was performed to quantify the net clinical benefit across a clinically relevant threshold probability range of 5%–30%, which corresponds to typical decision thresholds in PAH management.

#### External validation

The final model derived from the training cohort was assessed using an independent validation cohort (*n* = 98). All performance metrics (AUC, calibration statistics, Brier scores and DCA) were reported in the validation cohort to confirm the model’s transportability beyond the development dataset.

Additionally, to examine the predictive model’s performance robustness across demographic strata, subgroup analyses were performed in the validation cohort.

Statistical analyses were performed using SPSS (version 25.0, IBM, Armonk, New York) and R software (version 4.0.0). A *p* < 0.05 was considered statistically significant.
