# Supplementary material for: Development of a predictive model and nomogram using CTPA parameters for the identification of group 1 pulmonary arterial hypertension
Source: iScience. 2026 Oct 8;29(11):117763. doi: 10.1016/j.isci.2026.117763 (PMC13677309; doi:10.1016/j.isci.2026.117763)
Supplement: Supplementary file 1 [file mmc1.pdf]

iScience, Volume *29*

Supplemental information

Development of a predictive model and nomogram using CTPA parameters for the identification of group 1 pulmonary arterial hypertension

Huaxin Yuan, Iokfai Cheang, Gengmin Liang, Xu Zhu, Yutong Xing, Wenting Li, Shengen Liao, Jian He, Yunshan Cao, Xinli Li, Yi Xu, and Yanli Zhou

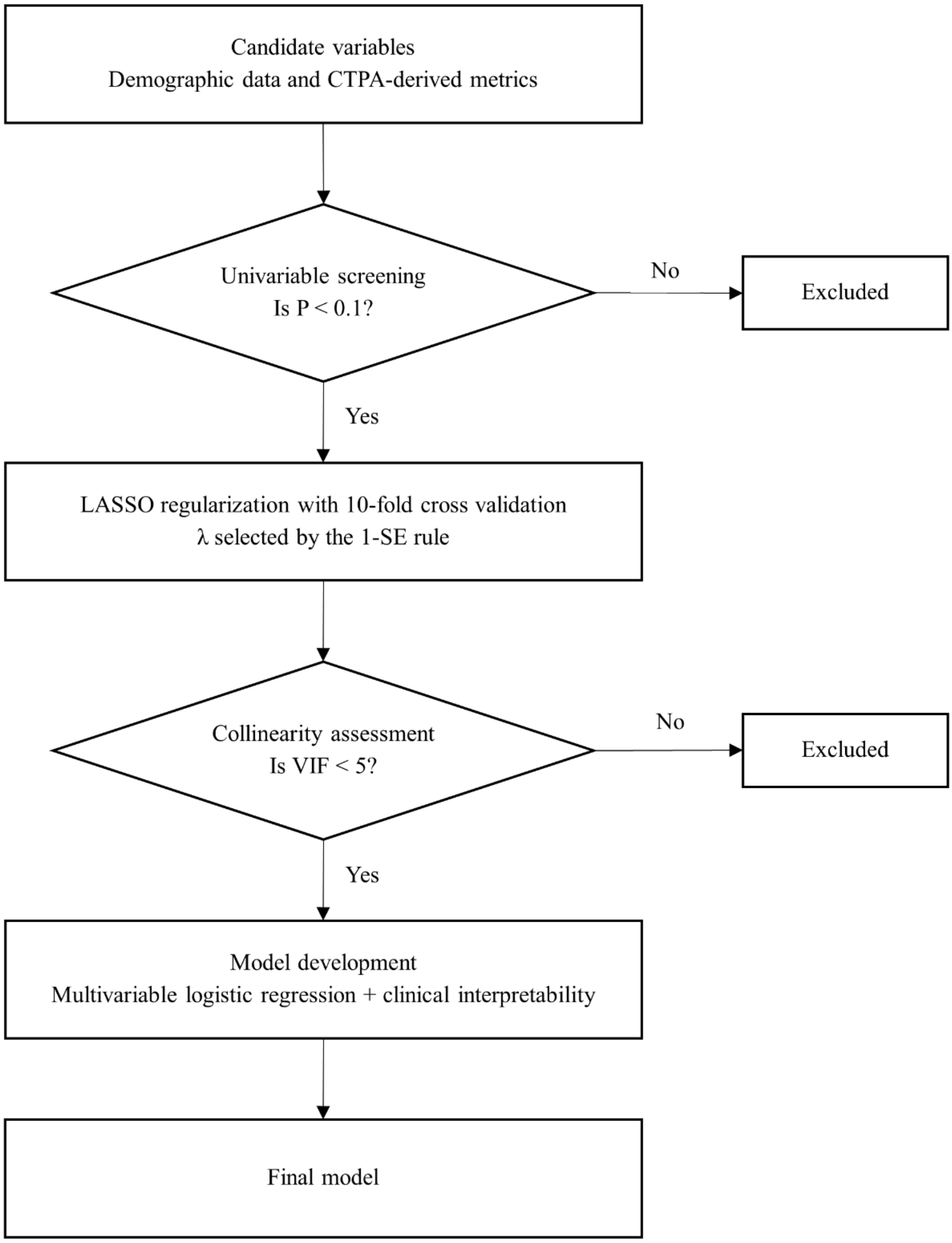

Figure S1. Flowchart of variable selection, Related to STAR Methods.
This flowchart illustrates the stepwise procedure for screening candidate variables and developing the final predictive model. CTPA, Computed tomography pulmonary angiography; LASSO, Least absolute shrinkage and selection operator; VIF, generalized Variance inflation factor; 1-SE, One-standard error.

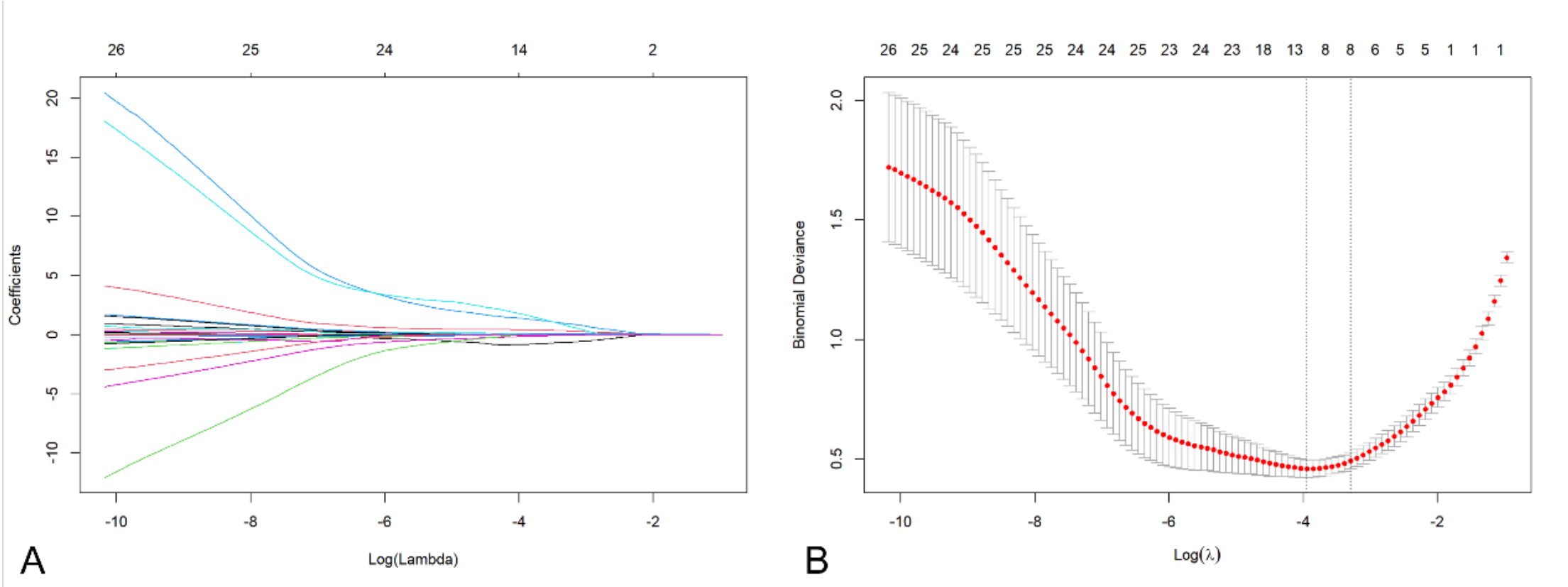

Figure S2. LASSO Regression Model, Related to STAR Methods.

(A) LASSO coefficient profiles. A coefficient profile plot was produced against the log (λ) sequence. (B) Different area under curve (AUC) values across the range of lambda. The AUC was estimated with 10-fold cross-validation, and the largest lambda value was chosen when the cross-validation error was within one standard error of the minimum.

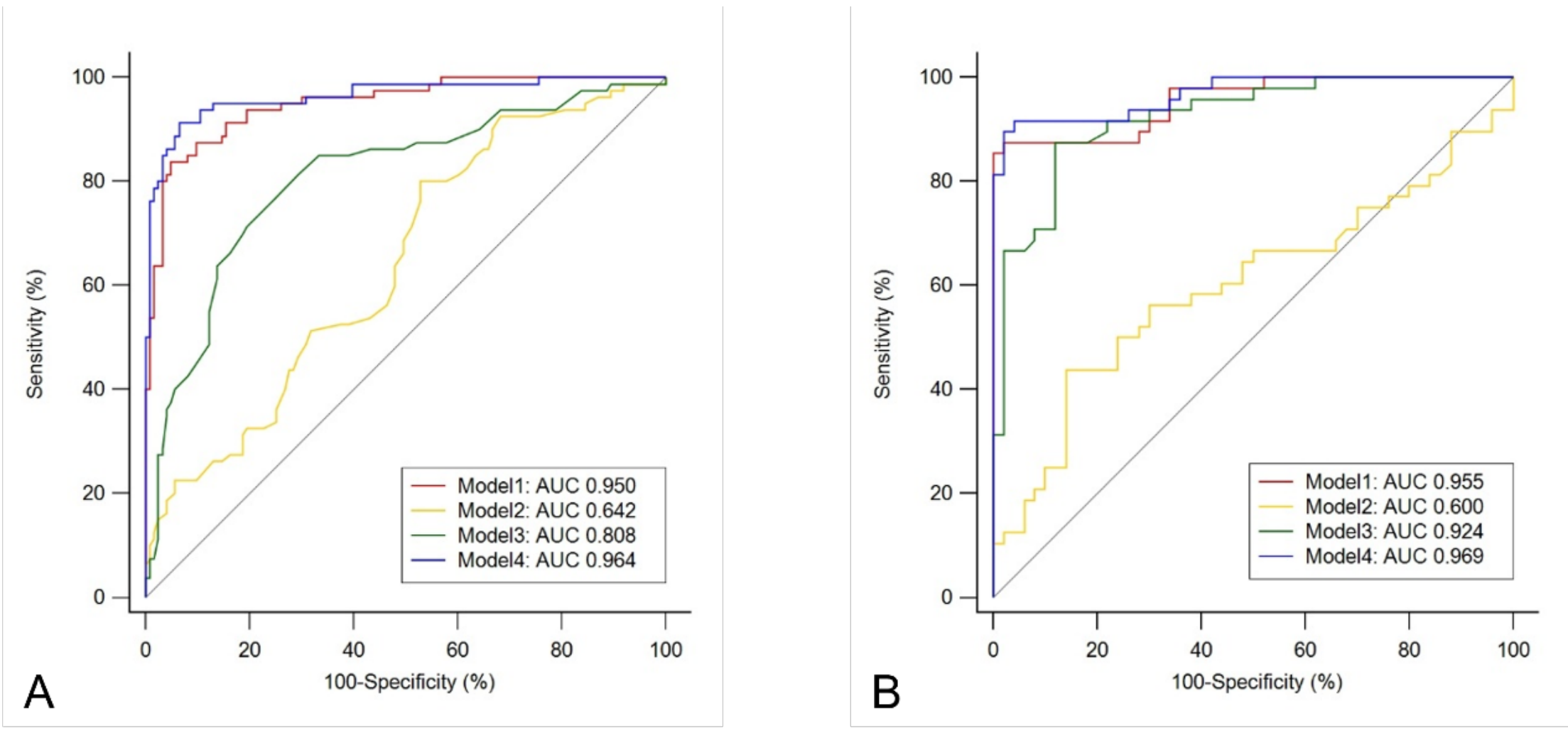

Figure S3. ROC curves comparing diagnostic performance of different models for PAH, Related to Figure 2 and 3.

The AUCs of these different models in the training cohort (A) and validation cohort (B): The red, yellow, green and blue line represents Model 1, Model 2, Model 3, Model 4 respectively. Model 1 included IVSA. Model 2 included IVST. Model 3 included RVOTW. Model 4 included IVSA, IVST and RVOTW. (Training Cohort: Model 1 vs Model 4, $P = 0.108$; Model 2 vs Model 4, $P = 0.001$; Model 3 vs Model 4, $P < 0.001$. Validation Cohort: Model 1 vs Model 4, $P = 0.182$; Model 2 vs Model 4, $P < 0.001$; Model 3 vs Model 4, $P = 0.035$.)

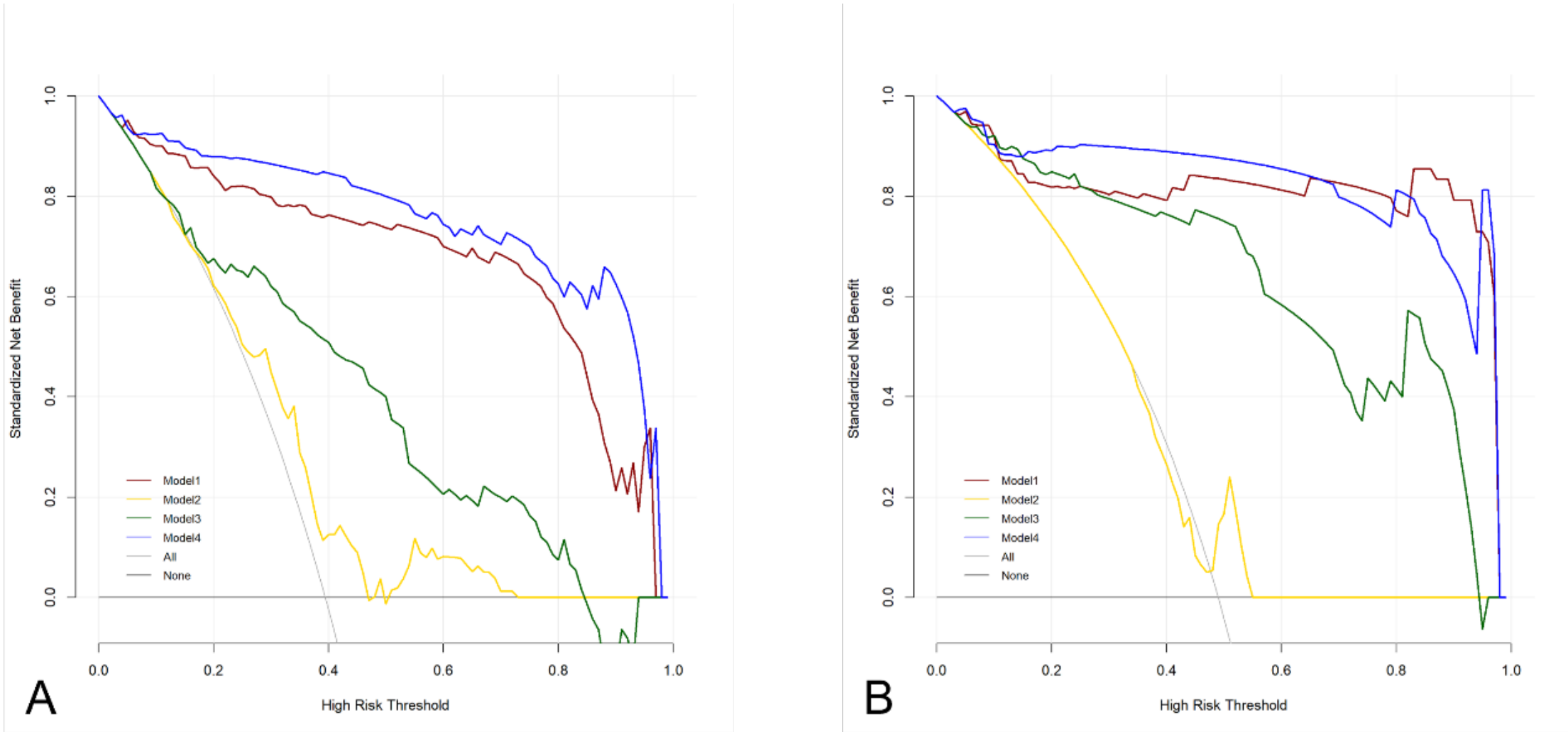

Figure S4. Decision curve analysis of these four models, Related to Figure 2 and 3.

DCA was performed in the training cohort (A) and validation cohort (B). The red, yellow, green and blue line represents Model 1, Model 2, Model 3, Model 4 respectively. The black line assumes no patients developed into PAH. The grey line assumes all subjects developed into PAH. Model 1 included IVSA. Model 2 included IVST. Model 3 included RVOTW. Model 4 included IVSA, IVST and RVOTW.

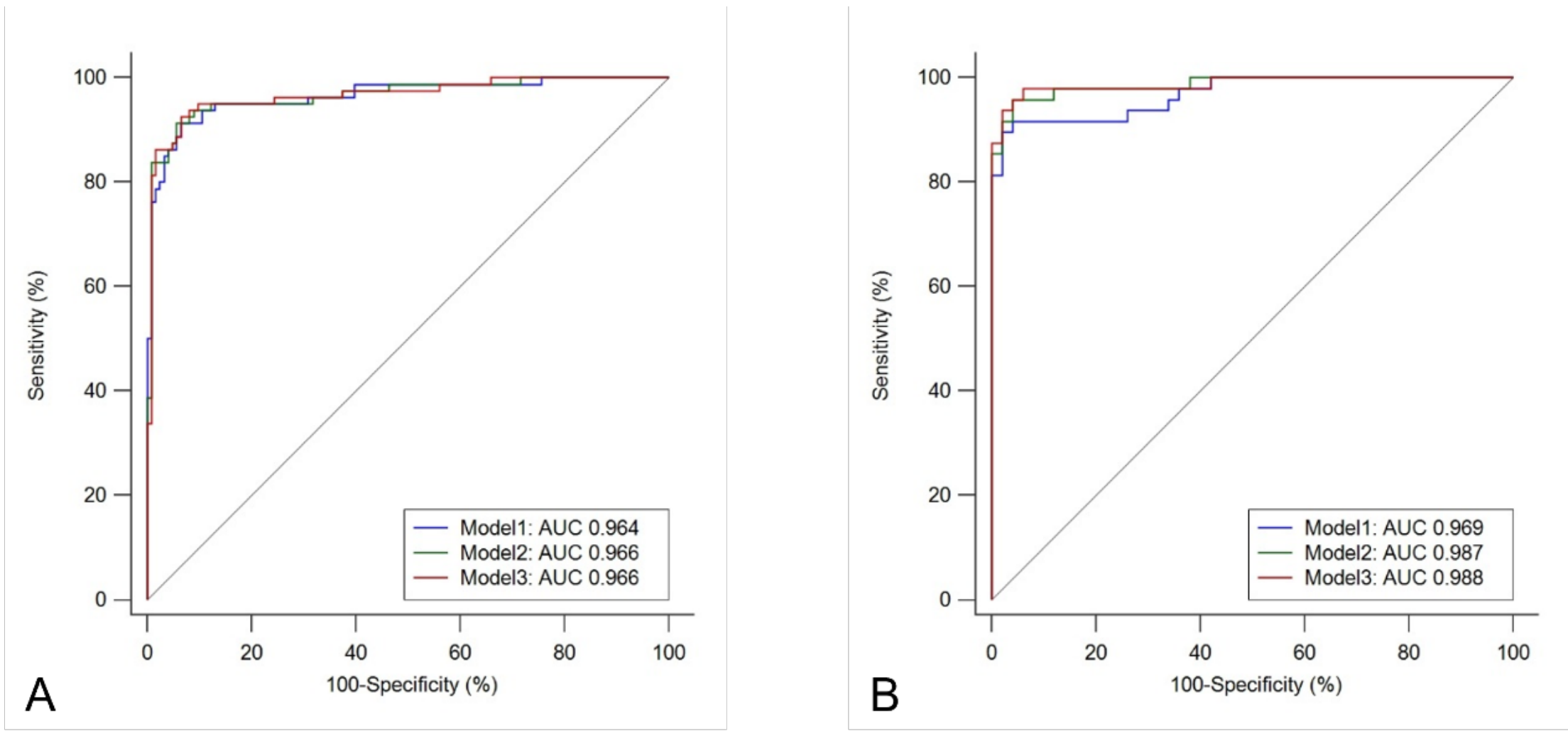

Figure S5. ROC Curves for these three models, Related to Results.

The AUCs of these three models in the training cohort (A) and validation cohort (B): The blue, green and red line represents Model 1, Model 2, Model 3 respectively. Model 1 included IVSA, IVST and RVOTW. Mode l 2 included model1 plus MPAd. Model 3 included model 1 plus rPA. (Training Cohort: Model 2 vs Model 1, P = 0.713; Model 3 vs Model 1, P = 0.681; Model 2 vs Model 3, P = 0.765. Validation Cohort: Model 2 vs Model 1, P = 0.101; Model 3 vs Model 1, P = 0.118; Model 2 vs Model 3, P = 0.660.)

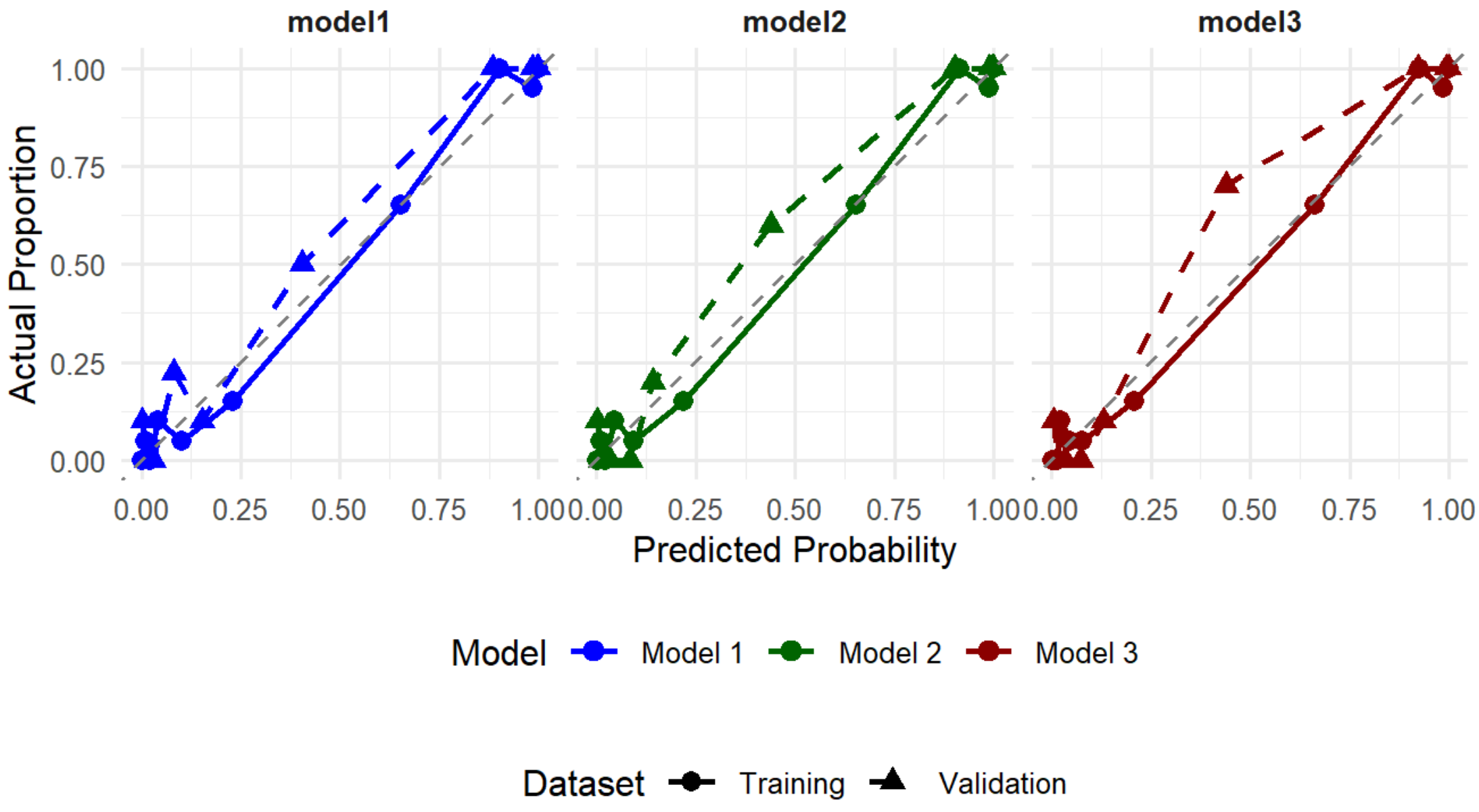

Figure S6. Calibration performance of three models on training and validation cohorts, Related to Results.

The dashed diagonal line represents the ideal calibration, where predicted probabilities perfectly match the observed events rates. Points falling below the diagonal indicate model overconfidence (predicted probability > actual proportion), whereas points above the line signify underconfidence (predicted probability < actual proportion). Model performance is evaluated on both training set (solid circles) and the independent validation set (solid triangles).

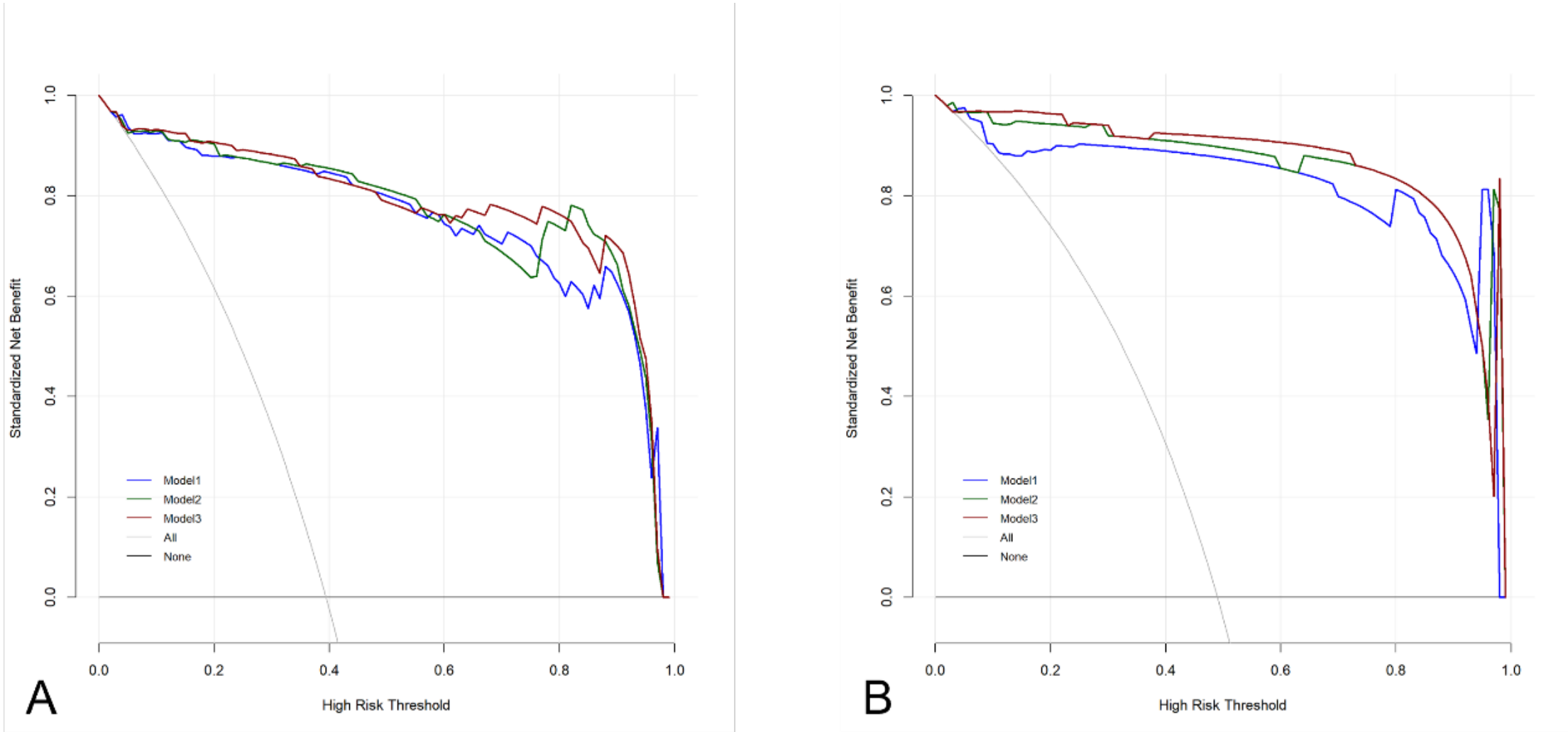

Figure S7. Decision curve analysis of these three models, Related to Results. DCA was performed in the training cohort (A) and validation cohort (B). The blue, green and red line represents Model 1, Model 2, Model 3 respectively. The black line assumes no patients developed into PAH. The grey line assumes all subjects developed into PAH. Model1 included IVSA, IVST and RVOTW. Model 2 included model1 plus MPAd. Model 3 included model 1 plus rPA.

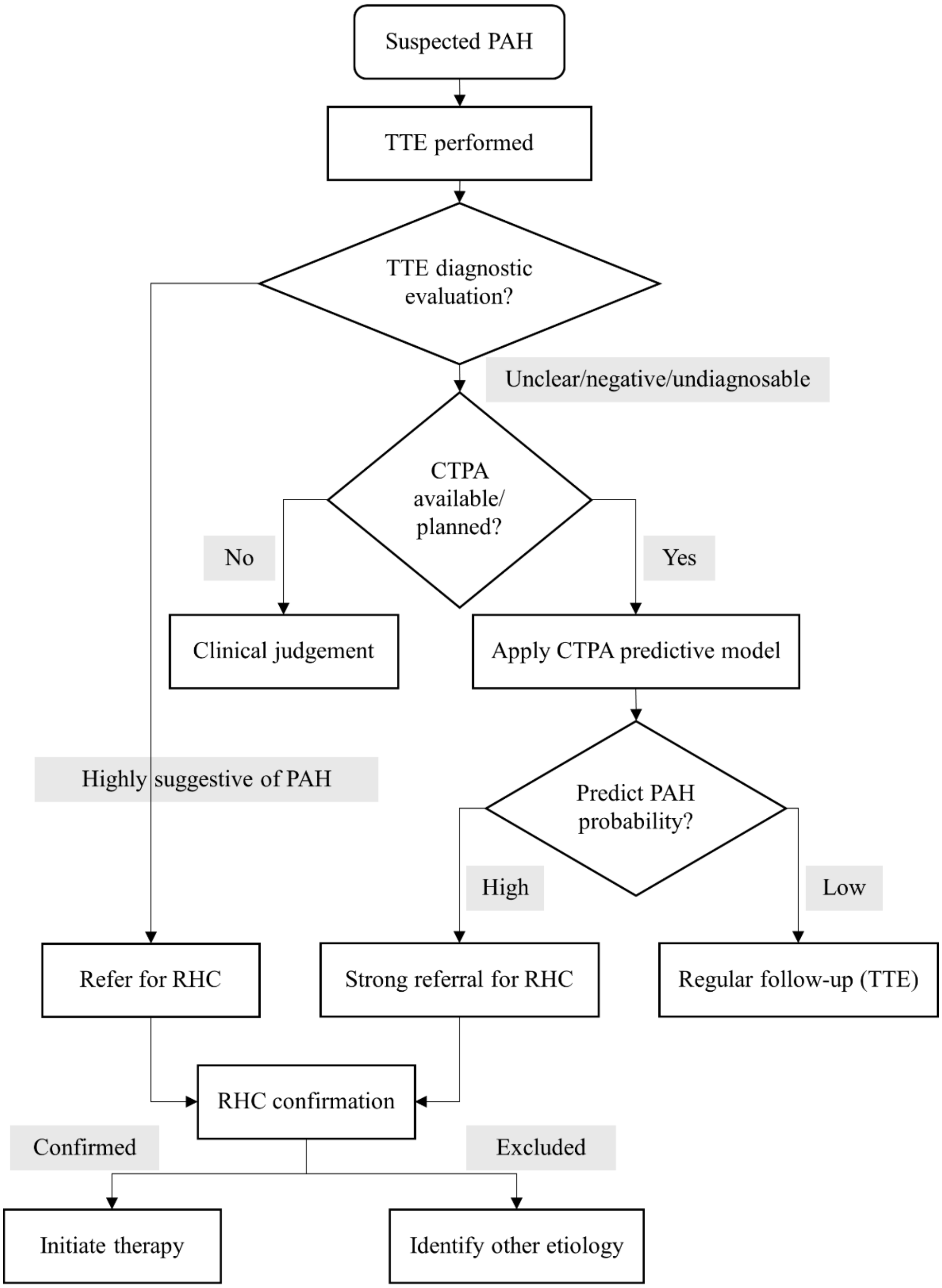

Figure S8. Stratified diagnostic algorithm for PAH, Related to Discussion.
This flowchart integrates conventional echocardiographic evaluation with a CTPA-based predictive model to guide RHC referral. PAH, Pulmonary arterial angiography; RHC, Right heart catheterization; TTE, Transthoracic echocardiography; CTPA, Computed tomography pulmonary angiography.

Table S1. Bootstrap internal validation results (1,000 resamples), Related to Results.

Metric	Training Cohort (n = 203)
AUC	0.964 (0.929-0.985)
Optimism-corrected AUC	0.960 (0.938-0.991)
Shrinkage Coefficient	0.993
Calibration Slope (95%CI)	1.000 (0.756-1.560)
Calibration Intercept (95%CI)	0.000 (-0.603-0.541)
Brier Score (95%CI)	0.062 (0.040-0.086)

Abbreviations: AUC, area under the receiver operating characteristic curve; CI, confidence interval.

Table S2. Comparative analysis of the diagnostic performance of models, Related to Figure 2 and 3.

Model	Training					Validation				
	Cutoff	AUC (95%CI)	Sensitivity (%)	Specificity (%)	P value	Cutoff	AUC (95%CI)	Sensitivity (%)	Specificity (%)	P value
**Model1**	137.68	0.950 (0.910-0.975)	83.75	95.12	<0.001	134.81	0.955 (0.894-0.987)	87.50	98.00	<0.001
**Model2**	10.50	0.642 (0.572-0.708)	80.00	47.15	<0.001	8.45	0.600 (0.497-0.698)	43.75	86.00	0.090
**Model3**	4.10	0.808 (0.747-0.860)	81.25	70.73	<0.001	4.60	0.924 (0.852-0.968)	87.50	88.00	<0.001
**Model4**	0.43	0.964 (0.929-0.985)	91.25	93.50	<0.001	0.30	0.969 (0.912-0.993)	91.67	96.00	<0.001

Note: Model 1 included IVSA. Model 2 included IVST. Model 3 included RVOTW. Model 4 included IVSA, IVST and RVOTW. IVSA, Interventricular Septal Angle; IVST, Interventricular Septal Thickness (mm); RVOTW, Right Ventricular Outflow Tract Wall thickness (mm).

Table S3. Subgroup analysis of model performance in the validation cohort, Related to Results.

Subgroup		n	PAH cases (%)	AUC (95% CI)	Sensitivity	Specificity
**Overall**		98	48 (49.0)	0.969 (0.912-0.993)	0.938	0.960
**Age**	< 60 years	57	38 (66.7)	0.981 (0.952-1.000)	0.947	1.000
	≥ 60 years	41	10 (24.2)	0.877 (0.689-1.000)	0.700	1.000
**Gender**	Female	65	33 (50.8)	0.955 (0.891-1.000)	0.909	1.000
	Male	33	15 (45.4)	0.963 (0.900-1.000)	0.867	1.000

Note: AUC, area under the receiver operating characteristic curve; CI, confidence interval; PAH, pulmonary arterial hypertension. Diagnostic threshold probability (Youden's index) = 0.404. Delong's test for AUC comparison: Age Subgroups D = 1.059, df = 41.892, p = 0.296; Gender subgroups D = 0.185, df = 85.908, p = 0.854.

Table S4. Variance inflation factor (VIF) values for variables in three Models, Related to Results.

Variable	Model 1	Model 2	Model 3
IVSA	1.244	1.423	2.092
IVST	1.037	1.078	1.040
RVOTW	1.209	1.827	1.355
MPAd	-	1.805	-
rPA	-	-	2.310

Note. A dash (-) indicates that the variable was not included in the model. VIF < 5 indicates no significant multicollinearity. Model 1 included IVSA, IVST and RVOTW. Model 2 included model 1 plus MPAd. Model 3 included model 1 plus rPA. MPAd, Main Pulmonary Artery diameter; AAd, Aortic Arch diameter; rPA, MPAd/AAd ratio.

Table S5. Incremental value of MPAd and rPA in the PAH prediction model, Related to Results.

Model	Training			Validation		
	AUC (95%CI)	Sensitivity (%)	Specificity (%)	AUC (95%CI)	Sensitivity (%)	Specificity (%)
**Model1**	0.964 (0.929-0.985)	91.25	93.50	0.969 (0.912-0.993)	91.67	96.00
**Model2**	0.966 (0.930-0.986)	91.25	94.31	0.987 (0.939-0.999)	95.83	96.00
**Model3**	0.966 (0.931-0.987)	92.50	93.50	0.988 (0.941-1.000)	97.92	94.00

Note. Model 1 included IVSA, IVST and RVOTW. Model 2 included model 1 plus MPAd. Model 3 included model 1 plus rPA. MPAd, Main Pulmonary Artery diameter; AAd, Aortic Arch diameter; rPA, MPAd/AAd ratio.

Table S6. Calibration performance of three models for PAH, Related to Results.

Model	Cohort	Slope (95% CI)	Intercept	Brier Score	Spiegelhalter P Value
**Model 1**	Training	1.000 (reference)	0.000	0.062	-
	Validation	0.946 (0.895-0.997)	0.520	0.055	0.136
**Model 2**	Training	1.000 (reference)	0.000	0.057	-
	Validation	1.177 (1.120-1.234)	0.749	0.047	0.200
**Model 3**	Training	1.000 (reference)	0.000	0.054	-
	Validation	1.219 (1.159-1.279)	0.839	0.044	0.204

Note: Model 1 included IVSA, IVST, and RVOTW. Model 2 added MPAd to Model 1, and Model 3 added rPA to Model 1. The training cohort results served as reference (slope=1, intercept=0 by definition). Lower Brier Scores indicate better overall predictive performance. Spiegelhalter's z-test for calibration in the validation cohort yielded p values of 0.136, 0.200, and 0.204 for Model 1, Model 2, and Model 3, respectively, indicating no significant departure from perfect calibration (all $p > 0.05$).

Table S7. Quantitative net benefit of three models and reference strategies at selected decision thresholds, Related to Results.

Decision Threshold	Model 1	Model 2	Model 3	Treat All	Treat None
**Training Cohort**					
0.05	0.372	0.371	0.371	0.362	0.000
0.10	0.356	0.357	0.359	0.327	0.000
0.15	0.354	0.358	0.360	0.287	0.000
0.20	0.355	0.356	0.358	0.243	0.000
0.25	0.343	0.346	0.355	0.192	0.000
0.30	0.336	0.346	0.348	0.134	0.000
**Validation Cohort**					
0.05	0.468	0.467	0.68	0.463	0.000
0.10	0.442	0.464	0.466	0.433	0.000
0.15	0.435	0.447	0.462	0.400	0.000
0.20	0.439	0.449	0.464	0.362	0.000
0.25	0.432	0.449	0.452	0.320	0.000
0.30	0.434	0.455	0.455	0.271	0.000

Note: Model 1 included IVSA, IVST and RVOTW. Mode l 2 included model1 plus MPAd. Model 3 included model 1 plus rPA. Net benefit values are rounded to three decimal places.
